# Role of Selected Organic Additives in Sulfate-Based Electroplating Baths for Copper Electrodeposition Toward Additive Manufacturing

**DOI:** 10.3390/molecules31101635

**Published:** 2026-05-13

**Authors:** Dawid Kiesiewicz, Karolina Syrek, Paweł Niezgoda, Maciej Pilch

**Affiliations:** 1 Faculty of Civil Engineering, Cracow University of Technology, Warszawska 24, 31-155 Cracow, Poland; 2 Faculty of Chemistry, Jagiellonian University, Gronostajowa 2, 30-387 Cracow, Poland; syrek@chemia.uj.edu.pl; 3 Faculty of Chemical Engineering and Technology, Cracow University of Technology, Warszawska 24, 31-155 Cracow, Poland

**Keywords:** sulfate-based electroplating baths, organic additives, copper electrodeposition, additive manufacturing

## Abstract

Precise control of copper electrodeposition is essential for electrochemical additive manufacturing based on layer-by-layer growth. In this work, the influence of selected organic additives, nicotinic acid, benzotriazole, thiourea and urea in sulfate-based electroplating baths was investigated with respect to their applicability in electrodeposition-driven 3D printing. Linear sweep voltammetry (LSV) was used to analyze the electrochemical behavior of Cu(II) reduction, while copper layers were deposited under potentiostatic conditions in a flow-assisted system (potential controlled conditions). The obtained deposits were characterized by SEM/EDS and quantitative measurements of layer thickness and dendrite height. The results show that the additives strongly affect both deposition kinetics and the morphology of electrodeposited layers. Benzotriazole acts as a strong inhibitor, producing fine-grained structures but reducing deposition efficiency and not fully suppressing vertical growth instabilities. Thiourea leads to highly unstable deposition with excessive dendritic growth and increased impurity incorporation. Nicotinic acid enables relatively thick deposits with moderate dendrite formation within an optimal concentration range. In contrast, urea provides the most stable growth, yielding uniform layers with minimal dendritic development and high copper purity. The dendrite height-to-layer thickness ratio proved to be an effective descriptor of electrodeposition growth stability. These findings highlight the critical role of additive selection in optimizing electroplating baths for electrochemical 3D printing applications.

## 1. Introduction

Hybrid metal–polymer materials have attracted growing interest because they combine the mechanical strength, electrical conductivity and thermal stability of metals with the low density, processability and geometric freedom of polymers [1,2,3,4]. Such systems are increasingly considered for lightweight structural components, embedded functional elements and integrated devices. However, their conventional fabrication still relies predominantly on multistep routes, such as joining, lamination, coating or secondary assembly, which limit both shape complexity and manufacturing integration [2,4].

Additive manufacturing (AM) offers a fundamentally different strategy by enabling direct layer-by-layer fabrication of complex architectures from digital models [5,6,7]. Over the past decade, polymer-based AM technologies have developed rapidly, particularly in the field of vat photopolymerization and extrusion-based printing, due to their high resolution, broad materials compatibility and relatively mild processing conditions [8,9,10,11,12,13]. In contrast, metallic AM remains dominated by powder-bed fusion and related thermal methods, which require high energy input, elevated temperatures and material-specific infrastructure [6,7]. This creates a strong incentive to develop alternative low-temperature routes for metal deposition that would be more compatible with hybrid metal–polymer manufacturing.

Electrodeposition is particularly promising in this context because it enables metal growth under ambient or near-ambient conditions with direct control over the process via potential, current density, electrolyte composition and transport regime [14,15,16,17]. From a manufacturing perspective, electrodeposition offers several attractive features, including relatively simple equipment, low thermal load and the possibility of spatially and temporally controlled material build-up. Importantly, electrodeposition-based 3D printing of metallic microarchitectures has already been demonstrated, confirming that electrochemical growth can be adapted from conventional plating to additive manufacturing concepts [14].

Copper is an especially relevant material for such studies because of its high electrical conductivity, widespread technological importance and well-established sulfate electrochemistry [15,16,17,18]. Nevertheless, copper electrodeposition under conditions favorable for rapid material build-up is highly sensitive to morphological instability. At sufficiently high cathodic polarization, deposition tends to become non-uniform due to local current-density amplification, concentration gradients and partial depletion of Cu^2+^ ions near the electrode surface [17,18]. As a consequence, nodular and dendritic structures can develop, especially at edges and protrusions, which is detrimental to dimensional accuracy, surface homogeneity and process reproducibility. These limitations are particularly critical in additive manufacturing, where structural defects generated in one deposition step may propagate through subsequent layers [19,20,21,22,23,24].

This issue is particularly important in the broader context of hybrid manufacturing strategies that combine electrochemical metal deposition with photopolymerization-based 3D printing. During the fabrication of such advanced hybrid components, in which both the polymer parts and the metallic parts possess strictly defined geometries and are directly joined during the additive manufacturing process, control of the morphology quality of both the deposited metal and the polymer is crucial, as it affects not only the strength and properties of each material individually, but also their mutual interaction (i.e., the adhesion strength between the polymer and the metal). Recent advances in visible light photoinitiating systems, photocatalysts and photocurable resin formulations have significantly expanded the possibilities of polymer-side processing in hybrid additive systems [25,26,27,28]. Consequently, the optimization of copper electrodeposition under conditions compatible with future integration into a coupled metal–polymer manufacturing platform becomes an important research objective.

Modern copper electroplating baths typically contain organic additives that act as suppressors, brighteners, accelerators or levelers [15,16,23,24]. Although organic additives used in copper electroplating baths are frequently grouped under the general term “brighteners”, their actual electrochemical functions may differ substantially depending on their adsorption behavior, interaction with copper ions and influence on interfacial charge-transfer processes. In practice, these additives may act as suppressors, corrosion inhibitors, grain refiners, levelers or adsorption regulators rather than as classical brighteners in the strict electroplating sense [15,16,24]. Their influence on copper electrodeposition is commonly associated with adsorption at the cathode/electrolyte interface, modification of the electrical double layer, alteration of Cu^2+^ reduction kinetics and regulation of nucleation and crystal growth mechanisms [16,18,24].

Benzotriazole (BTA) is generally regarded primarily as a corrosion inhibitor and suppressing additive rather than a conventional brightener. Its mechanism of action is associated with strong chemisorption on copper surfaces and the formation of Cu(I)-benzotriazole surface complexes or polymer-like Cu–BTA protective films [29,30,31]. These adsorbed layers partially block electrochemically active sites and hinder electron transfer between the cathode and copper ions in solution, thereby increasing cathodic polarization and reducing the effective deposition rate [29,31]. Because adsorption occurs preferentially at high-energy surface sites, such as protrusions or defect regions, BTA may also reduce local current-density amplification and contribute to grain refinement and smoother deposits [19,29]. However, excessive surface coverage may lead to overly strong suppression of copper reduction, resulting in increased overpotential and reduced deposition efficiency, particularly under high-current or mass-transport-limited conditions relevant to additive manufacturing [16,24].

Thiourea exhibits considerably more complex electrochemical behavior due to the presence of sulfur and its ability to undergo surface reactions during electrodeposition [20,32,33,34,35,36]. At relatively low concentrations, thiourea may adsorb on the cathode surface and modify nucleation kinetics, promoting the formation of finer grains and locally accelerating copper deposition through sulfur-containing intermediate species [20,32]. This effect has often been associated with transient activation of selected nucleation sites and modification of the earliest stages of electrocrystallization [32,33]. However, thiourea may also undergo electrochemical decomposition and generate adsorbed sulfur-containing species, which can alter the composition and stability of the growing deposit [34,35,36]. At higher concentrations, competitive adsorption, surface poisoning and incorporation of sulfur-containing reaction products may occur simultaneously, leading to unstable growth conditions, increased roughness and enhanced dendritic development [20,35]. Consequently, thiourea is not considered a simple suppressing additive, but rather a highly surface-active compound capable of producing both accelerating and inhibiting effects depending on concentration, local potential and hydrodynamic conditions.

Urea, in contrast, is generally regarded as a relatively weakly adsorbing additive whose role is more closely related to regulation of the interfacial electrochemical environment than to direct suppression of copper reduction [37,38,39,40]. Its mechanism is typically associated with weak adsorption through amino and carbonyl functional groups, modification of hydrogen-bonding interactions in the near-electrode region and possible weak complexation interactions with Cu^2+^ ions [37,38]. Unlike strongly adsorbing inhibitors, urea does not usually form compact blocking layers on the copper surface and therefore does not substantially suppress cathodic current [37,40]. Instead, it may contribute to stabilization of deposition conditions by moderating local concentration gradients and reducing fluctuations in interfacial charge-transfer behavior [40]. Such relatively mild and spatially homogeneous interactions may improve deposit uniformity without strongly decreasing the deposition rate, which is particularly advantageous for flow-assisted electrochemical additive manufacturing processes.

Nicotinic acid has been reported to influence copper electrodeposition mainly through adsorption-mediated modification of cathodic polarization and electrocrystallization behavior [18,24,41]. Owing to the presence of both pyridine and carboxylic functional groups, nicotinic acid may interact with the copper/electrolyte interface through coordination-type interactions and adsorption on active growth sites [18,41]. Such adsorption partially inhibits unrestricted crystal growth and modifies nucleation dynamics, thereby contributing to more homogeneous deposition conditions [18,24,41]. In contrast to strongly suppressing additives such as benzotriazole, nicotinic acid typically produces a more moderate increase in polarization while still maintaining relatively efficient copper deposition kinetics. As a result, it may provide a favorable balance between deposition rate and suppression of morphological instabilities, which is particularly important in electrodeposition-driven additive manufacturing systems operating under flow conditions.

Consequently, although benzotriazole, thiourea, urea and nicotinic acid are often collectively discussed as electroplating additives, their mechanisms of action differ fundamentally in terms of adsorption strength, surface reactivity, influence on nucleation and effect on copper growth stability. Therefore, they should not be regarded as functionally equivalent “brighteners”, but rather as additives exhibiting distinct electrochemical and microstructural roles during copper electrodeposition.

More generally, dendritic growth in copper deposition is strongly influenced by the interplay between interfacial kinetics, electrolyte composition and mass transport and may become dominant once local depletion and field focusing are not sufficiently compensated [21,22].

Although the role of additives in conventional copper electroplating has been widely investigated, their significance in electrodeposition-driven additive manufacturing is still not fully understood. This is especially true for systems operating under non-classical hydrodynamic conditions, where electrolyte renewal, convective transport and local adsorption–desorption equilibria may differ substantially from stagnant laboratory tests. In such cases, the additive should not merely inhibit deposition, but should selectively moderate it, preserving a sufficiently high deposition rate while minimizing roughness and dendritic growth [18,21,24].

Therefore, the aim of this study is to evaluate the influence of selected organic additives on copper electrodeposition from sulfate-based electroplating baths and to relate their electrochemical action to the morphology and microstructural quality of the resulting deposits. Particular attention is focused on the balance between electrodeposition rate and suppression of growth instabilities, since this balance is crucial for the future development of electrodeposition-assisted additive manufacturing of hybrid metal–polymer structures. It is worth emphasizing that the novelty of the results presented in this work also arises from the design of the applied copper electrodeposition system itself, which precisely reproduces the electrodeposition conditions occurring in the multimaterial 3D printer developed by the authors of the present article for the fabrication of hybrid metal–polymer components, constituting the subject of Polish Patent Office application no. P.452785 dated 28 July 2025.

## 2. Experimental Part

### 2.1. Materials

For preparing the electroplating baths, the following materials were used: copper(II) sulfate pentahydrate (Sigma-Aldrich, St. Louis, MO, USA), sulfuric acid (98%, Warchem, Zakręt, Poland) and brightening agents such as nicotinic acid (Sigma-Aldrich), urea (Sigma-Aldrich), thiourea (Sigma-Aldrich) and benzotriazole (Sigma-Aldrich).

### 2.2. Apparatus

The synthesized electroplating solutions were analyzed electrochemically by means of linear sweep voltammetry using a PalmSens4 potentiostat (PalmSens BV, Houten, The Netherlands). Measurements were conducted with a glassy carbon working electrode MF-2012 (3.0 mm diameter, BASi, West Lafayette, IN, USA), a platinum spiral counter electrode MW-1033 (BASi) and an Ag/AgCl reference electrode MF-2058 (BASi).

Copper electrodeposition experiments were performed in a custom-built system comprising two parallel electrodes: 1 cm^2^ copper cathode and 1.5 cm^2^ platinum anode (Supplementary Materials, Section S4, Figure S8). The electrodes were connected to a DC power source integrated with a potentiostat and immersed in the electrolyte contained within a reservoir. A programmable laboratory power supply ODP3122 Owon 30 V 12 A (OWON, Zhangzhou, China) was employed, supplemented with a custom-designed potentiostat intended for future integration with the developed electrodeposition-based metal 3D printing device. The reservoir was fitted with two hose connectors located on opposite side walls and linked via silicone tubing to a circulation pump, ensuring continuous electrolyte flow at approximately 6 L/min. The connectors were positioned so that the electrolyte stream passed directly through the inter-electrode region. The spacing between the electrodes was maintained at 1 cm. This configuration enabled preliminary replication of the electrodeposition conditions relevant to metal additive manufacturing. Such an approach was essential to ensure that the findings related to feedstock formulation for electrodeposition-driven 3D printing can be effectively translated into optimization of the actual printing process, which constitutes the central innovation of this work.

The morphology of the electrodeposited material, including both cross-sectional structure and surface features, was examined using a field-emission scanning electron microscope (FE-SEM/EDS, Hitachi S-4700 equipped with a Noran System 7, Hitachi High-Technologies Corporation, Tokyo, Japan).

Macroscopic images of the deposited copper layers were acquired with an optical microscope DSX1000 produced by OLYMPUS (Tokyo, Japan). The applied objective lens provided a magnification of 10× for the analyzed sample surface. Additionally, the thickness of the electrodeposited copper layers and the dendrite height were measured using a micrometer screw gauge (HAAS DIGITAL MICROMETER IP65, 0–25 mm, HAAS, Oxnard, CA, USA).

### 2.3. Methodology

#### 2.3.1. Linear Voltammetry

The measurement cell was first filled with the analyzed electroplating solution and positioned within the experimental setup. All electrodes were then introduced into the electrolyte, ensuring identical immersion depth for each of them. Subsequently, both mechanical stirring and argon purging were applied for 5 min. After this conditioning step, stirring and gas flow were discontinued and the measurement procedure was initiated. The investigated potential window ranged from 0 to −1.1 V versus the Ag/AgCl reference electrode, with a constant scan rate of 10 mV/s used in all experiments. Following each measurement, the working electrode surface was mechanically polished using diamond suspensions with grain sizes of 3 µm and 1 µm. Voltammetric experiments were conducted in electroplating baths prepared as saturated copper sulfate solutions containing a 1 M pH regulator and varying concentrations of brightening additives. The primary objective of these measurements was to enable qualitative comparison between different electrolyte compositions, rather than to provide a detailed kinetic evaluation.

#### 2.3.2. Electrodeposition of Copper Layers

Copper substrates with an active surface area of 1 cm^2^ were used as cathodes for electrodeposition in the prepared sulfuric acid–based electrolytes containing pH regulator, along with selected brightening additives. The process was conducted for 1 h. Deposition was carried out under potentiostatic conditions (constant potential control). During the experiments, the recorded current ranged from 0.3 to 0.5 A depending on the stage of deposition, yielding an estimated current density of about 0.3–0.5 A/cm^2^ for a cathode area of 1 cm^2^. The system operated in a three-electrode configuration (Ag/AgCl reference electrode, platinum anode, copper cathode) under flow conditions, with an electrolyte circulation rate of 6 L/min. The resulting samples consisted of copper layers deposited on square cathode substrates (1 cm × 1 cm) with variable thicknesses, as detailed in the Supplementary Materials. Preliminary tests (not included in this study) indicated the occurrence of gas bubble accumulation on electrode surfaces. However, in the experiments presented here, the introduction of electrolyte flow at a relatively high rate (6 L/min) effectively eliminated this issue. Under flow conditions, gas bubbles were rapidly removed by the flowing electrolyte, preventing their buildup on the electrodes. After electrodeposition, the samples were rinsed with distilled water followed by methyl alcohol.

Brightener concentrations and electrodeposition potentials were selected individually for each type of electroplating bath analyzed. Detailed information regarding the selection of brighteners can be found in the Supplementary Materials (Section S5). The selected conditions for each electroplating bath are presented in Table 1.

#### 2.3.3. SEM/EDS

SEM images were acquired using an accelerating voltage of 20.0 kV, with the working distance maintained at approximately 13.2–13.7 mm. SEM images were acquired at magnifications of approximately ×30 and ×250. Surface images were recorded with a 200 µm scale bar, whereas cross-sectional images were recorded with scale bars of 2.0–2.5 mm.

## 3. Results

### 3.1. Voltammetry

In order to determine the optimal voltage for conducting the copper electrodeposition process from electroplating baths containing different brightener additives, the current–voltage characteristics of these baths were determined using the linear voltammetry technique.

**Figure 1:**
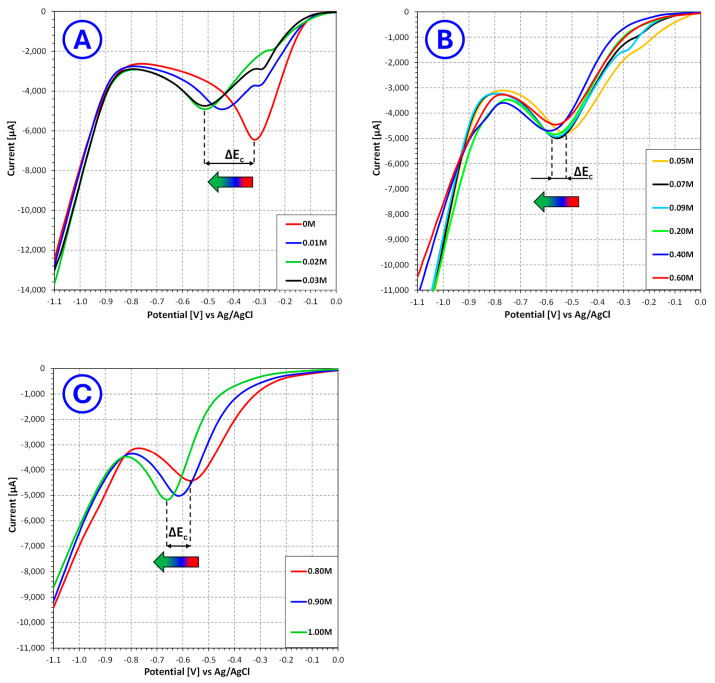
Influence of **nicotinic acid** concentration on the current–voltage characteristics of copper sulfate and sulfuric acid-based electroplating baths for selected **nicotinic acid** concentrations ranging from 0 mol/L to 0.03 mol/L (**A**), from 0.05 mol/L to 0.60 mol/L (**B**) and from 0.80 mol/L to 1.00 mol/L (**C**), where $\Delta E_{C}$—change in the potential of the cathodic signal minimum [V].

The LSV measurements demonstrated that **nicotinic acid** significantly affects the cathodic response of Cu(II) reduction in sulfate electroplating baths, with the magnitude and character of this effect strongly dependent on additive concentration. The analyzed voltammograms (Figure 1) were therefore divided into three concentration ranges: 0–0.03 mol/L, 0.05–0.60 mol/L and 0.80–1.00 mol/L, allowing the progressive evolution of the electrochemical response to be more clearly identified.

In the low concentration range (0–0.03 mol/L), only limited changes in the overall shape of the cathodic curves are observed. The voltammograms remain relatively similar in profile, which indicates that at these concentrations nicotinic acid does not yet induce a radical change in the reduction mechanism. At the same time, the minimum of the cathodic signal shifts gradually toward more negative potentials, which points to the onset of an inhibitory effect associated with adsorption of additive molecules at the cathode/electrolyte interface. This behavior suggests that even small amounts of nicotinic acid begin to hinder copper ion discharge, but the surface coverage is still too low for this effect to become strongly structure directing. From the viewpoint of electrochemical 3D printing, such concentrations are likely insufficient to effectively suppress local current-density amplification and dendritic protrusion growth.

A substantially clearer effect appears in the intermediate concentration range (0.05–0.60 mol/L). In this region, the cathodic signal minimum continues to shift toward more negative potentials, but the changes become more systematic and are accompanied by relatively moderate variation in the current value. This indicates that the additive increasingly modifies the interfacial kinetics without causing abrupt destabilization of the reduction process. Such behavior is characteristic of controlled adsorption, where the additive partially blocks the most active growth sites, thereby reducing the tendency toward uncontrolled crystal growth while still permitting efficient metal deposition. For additive manufacturing, this concentration range is particularly important, because it suggests the establishment of a more favorable balance between deposition rate and microstructural quality. In practice, this means that the nucleation and growth of copper may proceed in a more homogeneous way, which is beneficial for formation of compact layers and for reducing the probability of dendritic defects during layer-by-layer additive manufacturing.

The behavior changes again in the high concentration range (0.80–1.00 mol/L). Here, the minimum cathodic potential becomes markedly more negative, while the current response no longer follows the beneficial trend observed at intermediate concentrations. Instead, the data indicate stronger inhibition of the cathodic process, consistent with excessive adsorption of nicotinic acid on the electrode surface. Under such conditions, the additive most likely blocks a significant fraction of electrochemically active sites, increasing the overpotential required for copper deposition and lowering the practical deposition efficiency (deposition rate). Although strong inhibition may reduce the risk of rapid dendritic growth, it is not necessarily advantageous for electrochemical 3D printing, because excessively suppressed deposition can limit layer growth rate, decrease process productivity and make the system more sensitive to local disturbances in mass transport or field distribution.

**Figure 2:**
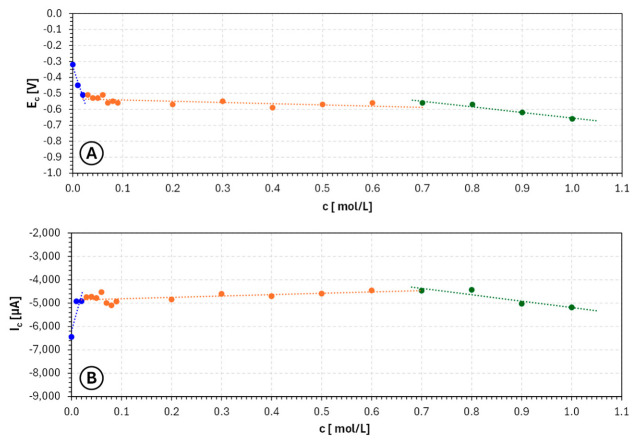
Dependence of cathodic signal potential (**A**) and current (**B**) on **nicotinic acid** concentration ranging from 0 mol/L to 1.00 mol/L for copper sulfate and sulfuric acid-based electroplating baths, where $E_{C}$—potential of the cathodic signal minimum [V] and $I_{C}$—current of the cathodic signal minimum [µA].

The concentration dependences summarized in Figure 2 confirm this interpretation. The potential corresponding to the cathodic signal minimum shifts overall toward more negative values with increasing nicotinic acid concentration, evidencing a progressive increase in cathodic polarization. At the same time, the current at the signal minimum does not change monotonically in a simple linear manner, but rather indicates the existence of an optimum concentration window in which inhibition is sufficient to stabilize the process without excessively reducing the deposition intensity. This is a particularly important observation from the perspective of electrochemical additive manufacturing, because an ideal electrolyte additive should not merely suppress deposition, but should selectively moderate it so as to promote uniform growth.

Overall, the LSV results indicate that nicotinic acid is a promising additive for copper electroplating baths intended for electrochemical 3D printing, but only within an appropriate concentration range. Very low concentrations appear too weak to substantially influence growth morphology, whereas very high concentrations lead to excessive cathodic inhibition. The most favorable range is the intermediate concentration domain, approximately 0.1–0.6 mol/L, where the electrolyte exhibits increased polarization accompanied by still relatively strong cathodic response. This suggests conditions under which dendritic growth may be reduced while maintaining sufficient deposition rate for practical manufacturing applications. From a technological perspective, the use of nicotinic acid in this intermediate range should favor more stable layer formation, improved geometric controllability of deposited copper and better compatibility with electrochemical 3D printing performed under flow conditions. Consequently, these concentrations may be regarded as the most suitable for further optimization studies involving actual deposition experiments, morphological characterization and assessment under modulated current or potential regimes.

**Figure 3:**
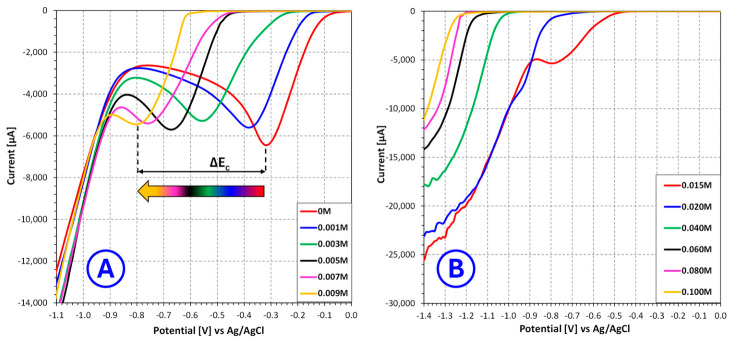
Influence of **benzotriazole** concentration on the current–voltage characteristics of copper sulfate and sulfuric acid-based electroplating baths for selected **benzotriazole** concentrations ranging from 0 mol/L to 0.009 mol/L (**A**) and from 0.015 mol/L to 0.100 mol/L (**B**), where $\Delta E_{C}$—change in the potential of the cathodic signal minimum [V].

The linear sweep voltammetry results indicate that **benzotriazole** strongly affects the electrochemical behavior of copper deposition in sulfate electroplating baths, with a markedly different character compared to nicotinic acid. Due to the relatively low concentration range in which benzotriazole is active, the analysis was divided into two regions: 0–0.009 mol/L and 0.015–0.100 mol/L, corresponding to the datasets presented in Figure 3A and Figure 3B, respectively.

In the low concentration range (0–0.009 mol/L), even small additions of benzotriazole lead to noticeable changes in the voltammetric response. A distinct shift of the cathodic signal minimum toward more negative potentials is observed, accompanied by a reduction in cathodic current. This behavior suggests that benzotriazole exhibits strong adsorption at the cathode surface even at low concentrations, forming a partially blocking layer that inhibits copper ion reduction. Such an effect is consistent with the known ability of benzotriazole to form surface complexes with copper, which can significantly alter interfacial charge-transfer processes [29,30,31]. The relatively rapid onset of this inhibition indicates that the additive has high surface activity and a strong affinity for the electrode surface.

In the higher concentration range (0.015–0.100 mol/L), the inhibitory effect becomes significantly more pronounced. The cathodic signal minimum shifts further toward negative potentials, while the current response is increasingly suppressed. In addition, changes in the shape of the voltammetric curves may be observed, which can indicate alterations in the mechanism of copper deposition. These effects may be attributed to the formation of a more continuous and compact adsorbed layer of benzotriazole or copper–benzotriazole complexes, effectively blocking a substantial fraction of active nucleation and growth sites. Under such conditions, the electrochemical reduction of copper ions becomes increasingly hindered, requiring higher overpotentials to proceed.

**Figure 4:**
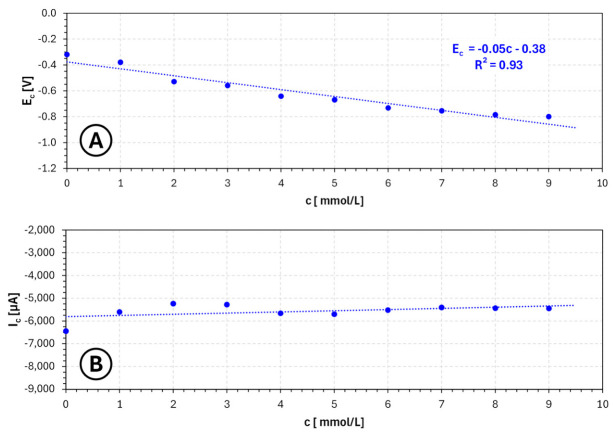
Dependence of cathodic signal potential (**A**) and current (**B**) on **benzotriazole** concentration ranging from 0 mol/L to 0.01 mol/L for copper sulfate and sulfuric acid-based electroplating baths, where $E_{C}$—potential of the cathodic signal minimum [V] and $I_{C}$—current of the cathodic signal minimum [µA].

The concentration dependencies summarized in Figure 4 further confirm these trends. The potential of the cathodic signal minimum exhibits a systematic shift toward more negative values with increasing benzotriazole concentration, reflecting progressive cathodic polarization. At the same time, the current at the signal minimum decreases significantly, indicating a reduction in the effective deposition rate. This combination of strong polarization and current suppression is characteristic of additives that act as efficient surface blockers.

From the perspective of electrochemical additive manufacturing, such behavior has important implications. On the one hand, the strong adsorption of benzotriazole may effectively suppress localized current density peaks and reduce the tendency for dendritic growth, which is beneficial for achieving smoother and more uniform deposits. On the other hand, excessive inhibition may lead to a substantial decrease in deposition rate and reduced process efficiency, which is undesirable for layer-by-layer fabrication.

Importantly, the results suggest that benzotriazole operates within a relatively narrow optimal concentration window. At very low concentrations, its effect may be insufficient to fully stabilize the growth process, whereas at higher concentrations, the deposition becomes strongly suppressed. Therefore, the most favorable conditions for practical applications are likely to be found at low to moderate concentrations (on the order of 0.001–0.01 mol/L), where the additive can partially inhibit the process without completely blocking electrochemical activity.

Within this range, benzotriazole may contribute to improved control over nucleation and growth processes, potentially leading to more uniform layer formation and reduced surface roughness. However, due to its strong inhibitory nature, careful optimization is required to avoid excessive polarization and loss of deposition efficiency. Consequently, benzotriazole appears to be a highly active but sensitive additive, whose applicability in electrochemical 3D printing depends critically on precise concentration control and may benefit from combination with other additives or the use of modulated deposition regimes.

**Figure 5:**
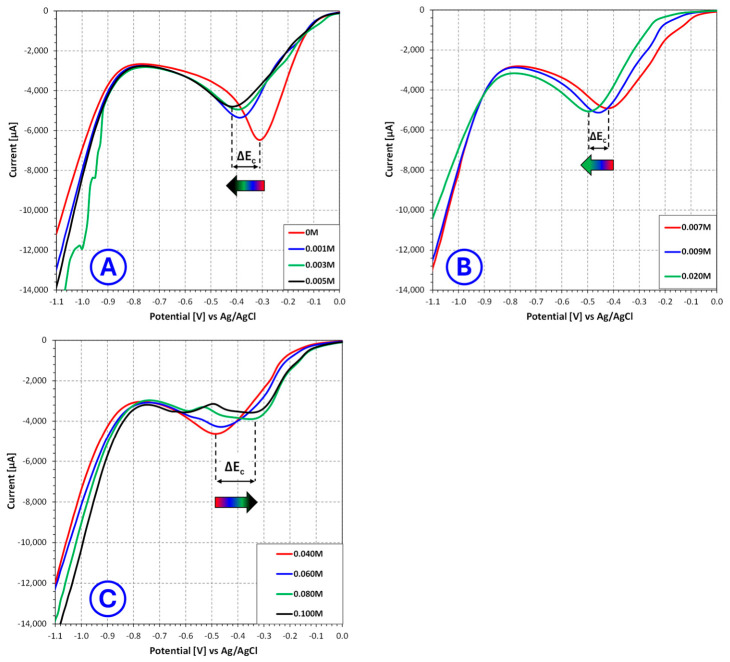
Influence of **thiourea** concentration on the current–voltage characteristics of copper sulfate and sulfuric acid-based electroplating baths for selected **thiourea** concentrations ranging from 0 mol/L to 0.005 mol/L (**A**), from 0.007 mol/L to 0.020 mol/L (**B**) and from 0.040 mol/L to 0.100 mol/L (**C**), where $\Delta E_{C}$—change in the potential of the cathodic signal minimum [V].

The linear sweep voltammetry results demonstrate that **thiourea** significantly modifies the copper electrodeposition, with a strongly concentration-dependent effect that differs in character from both nicotinic acid and benzotriazole. Due to the wide concentration range investigated, the analysis was divided into three regions: 0–0.005 mol/L, 0.007–0.020 mol/L and 0.040–0.100 mol/L, corresponding to Figure 5A–C, respectively.

In the low concentration range (0–0.005 mol/L), the addition of thiourea leads to noticeable changes in the cathodic response even at very small concentrations. A shift of the cathodic signal minimum toward more negative potentials is observed, accompanied by variations in the current response. This behavior suggests that thiourea readily adsorbs on the cathode surface and begins to influence the kinetics of copper ion reduction. However, unlike purely inhibiting additives, the effect of thiourea at low concentrations may involve both inhibition and localized acceleration phenomena, which are commonly associated with sulfur-containing organic compounds [20,32,33,34].

In the intermediate concentration range (0.007–0.020 mol/L), the voltammetric behavior becomes more irregular. Although the cathodic signal minimum continues to shift toward more negative potentials, the shape of the curves indicates less uniform behavior, with more pronounced deviations between individual concentration levels. This may suggest the coexistence of competing processes, such as adsorption–desorption dynamics, surface complex formation and possible catalytic effects of intermediate species. Thiourea is known to undergo electrochemical transformations and to form surface-bound sulfur-containing species, which can modify both nucleation and growth mechanisms in a non-uniform manner [20,35,36]. Consequently, the system may exhibit increased sensitivity to local conditions at the electrode surface.

In the high concentration range (0.040–0.100 mol/L), the influence of thiourea becomes even more pronounced, leading to significant changes in both the position and shape of the voltammetric curves. The cathodic signal minimum is further shifted toward negative potentials, indicating increased polarization, while the current response shows signs of instability or non-monotonic behavior. These observations may reflect strong surface interactions combined with the formation of reaction intermediates or decomposition products that interfere with the copper deposition process. In this regime, the additive likely exerts both inhibitory and destabilizing effects, which may result in heterogeneous surface activity and uneven growth conditions.

**Figure 6:**
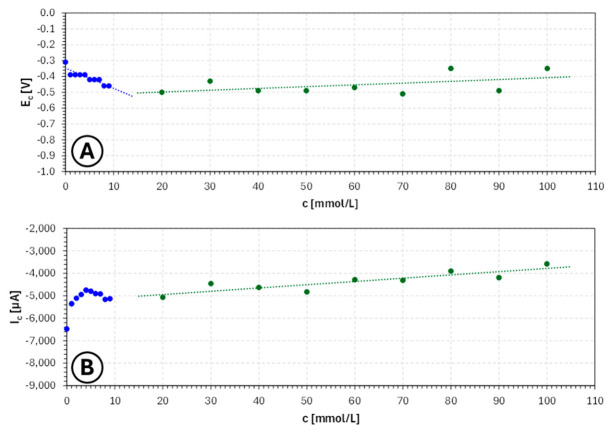
Dependence of cathodic signal potential (**A**) and current (**B**) on **thiourea** concentration ranging from 0 mol/L to 0.1 mol/L for copper sulfate and sulfuric acid-based electroplating baths, where $E_{C}$—potential of the cathodic signal minimum [V] and $I_{C}$—current of the cathodic signal minimum [µA].

The trends summarized in Figure 6 support this interpretation. The potential corresponding to the cathodic signal minimum generally shifts toward more negative values with increasing thiourea concentration, indicating progressive polarization of the system. However, the current at the signal minimum does not follow a simple monotonic trend and instead exhibits variability across the concentration range. Such behavior suggests that thiourea does not act as a simple inhibitor, but rather introduces competing effects that influence the electrochemical process in a more complex manner.

From the perspective of electrochemical additive manufacturing, these findings have important implications. While thiourea may, at low concentrations, contribute to modification of nucleation behavior, its tendency to induce non-uniform and potentially unstable electrochemical responses at higher concentrations is likely undesirable. The presence of competing adsorption and reaction processes may lead to localized variations in current density, which can promote uneven growth and increase the risk of defect formation, including nodular or dendritic structures.

Therefore, although thiourea can influence copper electrodeposition, its applicability as a process-stabilizing additive for electrochemical 3D printing appears limited. If used, it should be restricted to very low concentrations (below approximately 0.005 mol/L), where its impact on the system remains moderate. At higher concentrations, the observed electrochemical behavior suggests increasing instability and reduced controllability of the deposition process.

In summary, thiourea behaves as a highly active but complex additive, whose effects on copper electrodeposition are governed by a combination of adsorption, surface reactions and possible decomposition pathways. Unlike nicotinic acid, which promotes controlled inhibition or benzotriazole, which acts as a strong surface blocker, thiourea introduces a mixed and less predictable influence on the electrochemical process. As a result, its use in electrochemical additive manufacturing requires careful control and may not be optimal for applications requiring high process stability and reproducibility.

**Figure 7:**
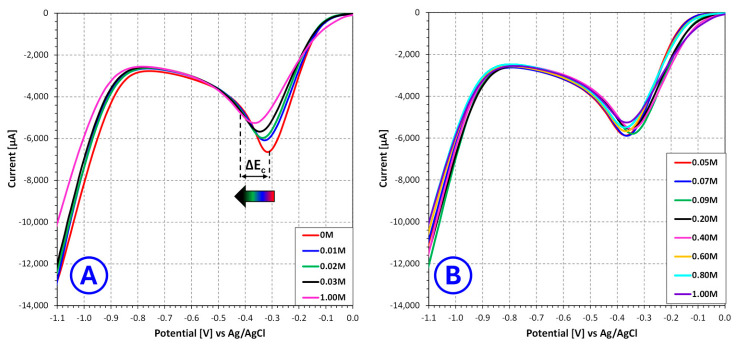
Influence of **urea** concentration on the current–voltage characteristics of copper sulfate and sulfuric acid-based electroplating baths for selected **urea** concentrations ranging from 0 mol/L to 1.00 mol/L (**A**) and from 0.05 mol/L to 1.00 mol/L (**B**), where $\Delta E_{C}$—change in the potential of the cathodic signal minimum [V].

The linear sweep voltammetry results indicate that **urea** influences the electrochemical behavior of copper deposition in sulfate electroplating baths in a relatively moderate and gradual manner compared to the other investigated additives. The analysis was conducted over a wide concentration range (0–1.00 mol/L), with representative datasets presented in Figure 7A,B.

At low concentrations (0–0.03 mol/L), the addition of urea results in only minor changes in the shape of the voltammetric curves. The cathodic signal minimum exhibits a slight shift toward more negative potentials, indicating a weak increase in cathodic polarization. This behavior suggests limited adsorption of urea molecules at the cathode surface and a relatively small influence on the charge-transfer kinetics of copper ion reduction. Unlike strongly adsorbing additives such as benzotriazole, urea does not appear to form a compact blocking layer and therefore its effect on the electrochemical process remains relatively subtle [37,38,39,40].

At higher concentrations (0.05–1.00 mol/L), the cathodic signal minimum continues to shift toward more negative potentials. However, the magnitude of this shift becomes less pronounced at higher concentrations. This behavior suggests that the influence of urea on the electrode surface may approach a saturation regime. At the same time, the current response exhibits only moderate variation and does not show pronounced suppression or a clear monotonic trend. This indicates that urea exerts a weak inhibitory effect without significantly hindering the electrochemical reduction process. Importantly, the voltammetric curves do not exhibit significant distortion in shape. This suggests that even at elevated concentrations, urea does not strongly block active sites or fundamentally alter the deposition mechanism.

**Figure 8:**
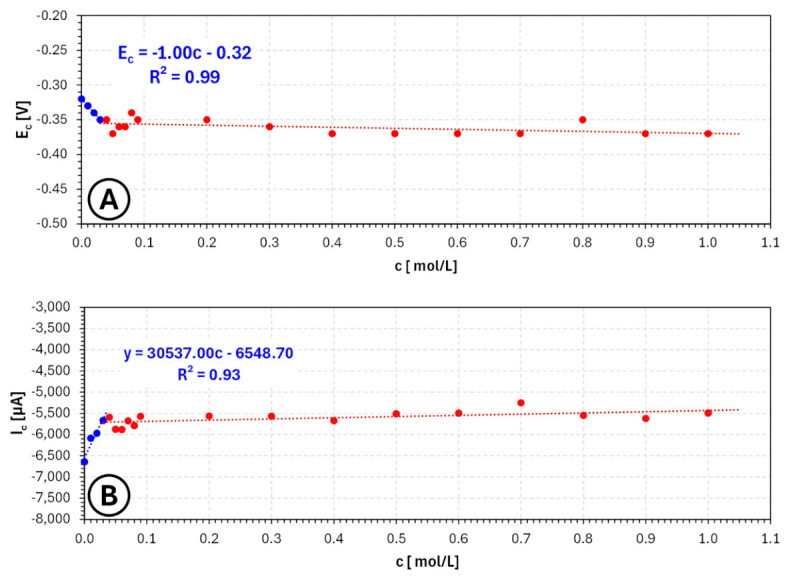
Dependence of cathodic signal potential (**A**) and current (**B**) on **urea** concentration ranging from 0 mol/L to 1.0 mol/L for copper sulfate and sulfuric acid-based electroplating baths, where $E_{C}$—potential of the cathodic signal minimum [V] and $I_{C}$—current of the cathodic signal minimum [µA].

The trends summarized in Figure 8 support this interpretation. The potential corresponding to the cathodic signal minimum shows an overall shift toward more negative values with increasing urea concentration, although this dependence is not strictly linear. At the same time, the current at the signal minimum varies within a relatively narrow range and does not exhibit a strong concentration-dependent trend. This behavior is characteristic of weakly interacting additives that modify the electrochemical environment without strongly interfering with charge-transfer processes.

From the perspective of electrochemical additive manufacturing, these characteristics are advantageous. The relatively mild influence of urea suggests that it can be used to fine-tune the deposition process without introducing significant instability or excessive inhibition. In contrast to benzotriazole, which strongly suppresses deposition or thiourea, which introduces complex and potentially unstable behavior, urea provides a more predictable and controllable modification of electrochemical conditions. Importantly, the absence of pronounced current suppression suggests that the deposition rate can be maintained across a wide concentration range, which is beneficial for process efficiency. At the same time, the moderate increase in polarization may contribute to improved uniformity of deposition by reducing the tendency for localized current density amplification. Therefore, urea may promote a more homogeneous growth regime without significantly compromising productivity. Overall, the results suggest that urea is a suitable additive for applications requiring stable and reproducible electrodeposition conditions. Unlike more strongly interacting additives, it does not exhibit a sharply defined optimal concentration window; instead, it can be applied over a relatively broad range (approximately 0.05–1.00 mol/L) without inducing detrimental electrochemical effects. From a technological standpoint, urea may therefore be particularly useful as a supporting or stabilizing additive in electrochemical 3D printing systems, where it can complement more active species by enhancing process stability and reducing sensitivity to local fluctuations. Its moderate influence and wide operating range make it a promising candidate for integration into multi-component electroplating bath formulations for additive manufacturing applications.

It should be noted that the presented voltammetric measurements were performed under stagnant (no-flow) conditions. However, under the flow regime characteristic of electrochemical 3D printing, it may be hypothesized that the inhibition mechanism remains adsorption controlled, while the effective surface coverage is influenced by the dynamic balance between adsorption, desorption and convective mass transport. Consequently, the magnitude and spatial uniformity of the inhibitory effect may differ from static conditions, particularly for weakly adsorbing additives, due to continuous electrolyte renewal at the electrode interface.

### 3.2. SEM Analysis

**Figure 9:**
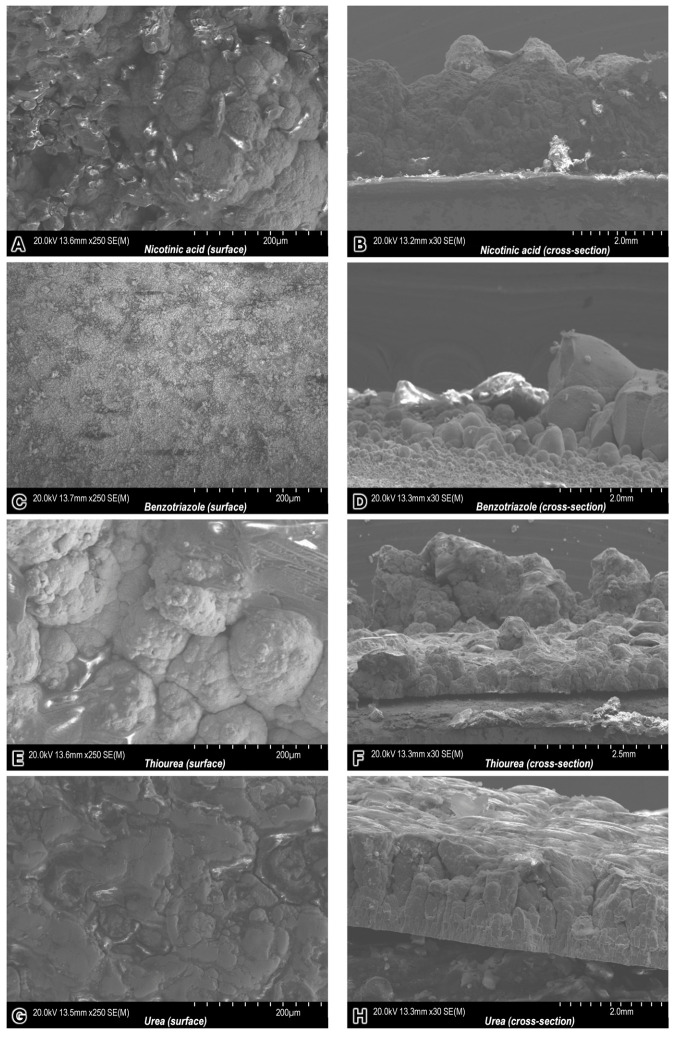
SEM images of the electrodeposited copper layers obtained using electroplating baths with various brighteners: nicotinic acid (**A**,**B**), benzotriazole (**C**,**D**), thiourea (**E**,**F**) and urea (**G**,**H**) on the surface of the sample (on the left side) and in its cross-section (on the right side).

Copper layers were electrodeposited from copper sulfate plating baths containing different brightening additives. Figure 9 presents SEM micrographs illustrating both the surface morphology and cross-sectional structure of the resulting deposits.

The SEM analysis of copper electrodeposits obtained from sulfate baths with different organic additives revealed pronounced differences in microstructure, grain size and growth stability.

In the case of nicotinic acid, the surface morphology was dominated by large, coalesced nodular structures with characteristic cauliflower-like features. The lateral size of these agglomerates was estimated to be on the order of ~80–250 µm. Cross-sectional observations indicated a relatively thick deposit (~2.37 mm), with surface protrusions reaching ~0.84 mm, corresponding to a dendrite-to-layer thickness ratio of ~0.35. The internal structure appeared relatively continuous, although locally heterogeneous, suggesting stable but diffusion-influenced growth.

For benzotriazole-containing electrolytes, a highly refined and homogeneous surface morphology was observed. The grain size was significantly reduced, with characteristic dimensions below ~5 µm, forming a compact, fine-grained structure. The cross-sectional images revealed a distinct bimodal morphology: a dense base layer topped with localized larger protrusions (~100–200 µm). The total layer thickness was approximately ~1.78 mm, while the protrusion height reached ~1.15 mm, resulting in a dendrite-to-thickness ratio of ~0.65. This indicates that, although benzotriazole promotes grain refinement, significant vertical growth instabilities may still occur.

A significantly different morphology was observed for thiourea. The surface consisted of very large, irregular agglomerates with dimensions exceeding ~150–300 µm, forming a highly rough and heterogeneous structure. The cross-sectional images confirmed a highly uneven layer (~1.34 mm thick) and very pronounced protrusions reaching ~1.78 mm. This corresponds to a dendrite-to-thickness ratio of ~1.33, the highest among all tested additives. The internal structure was highly porous and discontinuous, indicating strong local current density fluctuations and highly unstable growth.

The use of urea resulted in a comparatively compact and uniform morphology. The surface was composed of moderately sized grains (~20–80 µm) with limited agglomeration and significantly reduced roughness compared to thiourea. The cross-sectional structure revealed a dense morphology with relatively uniform thickness (~1.79 mm). Surface protrusions were limited to ~0.21 mm, yielding a dendrite-to-thickness ratio of ~0.12. The layer exhibited good continuity and low porosity, indicating a stable growth regime with effective suppression of dendritic development.

In summary, the results confirm that the type of organic additive has a decisive influence on both the electrodeposition kinetics and the surface morphology of copper layers. Benzotriazole provides strong grain refinement but does not fully suppress vertical growth instabilities. Thiourea leads to the most unstable growth, characterized by excessive dendritic development exceeding the layer thickness. Nicotinic acid shows moderate stability with substantial dendritic contribution, while urea ensures the most stable growth with minimal dendritic formation. The ratio of dendrite height to layer thickness proved to be a useful quantitative parameter, increasing in the following order: urea (~0.12) < nicotinic acid (~0.35) < benzotriazole (~0.65) << thiourea (~1.33), reflecting a transition from stable to highly unstable electrodeposition regimes.

The values of the thickness of the electrodeposited copper layers, dendrite heights and their corresponding ratios, determined based on SEM micrographs for samples obtained from electroplating baths containing various brightening additives, are summarized in Table 2. It should be noted that these values represent point measurements derived from individual cross-sectional SEM images of each sample. Consequently, they do not provide a comprehensive representation of the overall layer thickness or dendrite height, as these parameters may vary across different regions of the sample.

### 3.3. Layer Thickness and Dendrite Height Measurements

**Figure 10:**
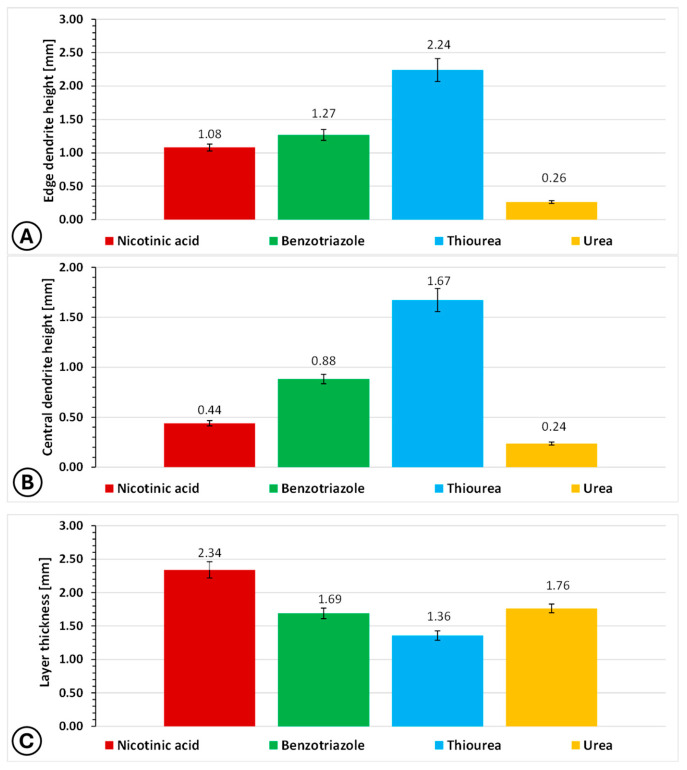
Comparison of representative average values of layer thickness (**A**), edge dendrite height (**B**) and central dendrite height (**C**) for copper layers obtained by electrodeposition from electroplating baths containing various brightening agents. Measurements were performed using micrometer screw at three measurement points. The plot presents mean values with standard deviation.

To determine representative average values of the layer thickness and dendrite height for each sample, measurements were carried out using a micrometer screw at three different locations per sample. The dendrite height measurements were performed separately for the edge regions and the central area of each sample, as it was observed that in some cases dendritic structures were predominantly present at the sample edges (Supplementary Materials, Section S2, Figures S1–S3). The average values of copper layer thickness, edge dendrite height and central dendrite height determined in this way are presented in Figure 10A–C.

The comparative analysis of layer thickness and dendrite height (Figure 10) indicates that the type of brightener has a decisive influence not only on the overall growth rate but also on the spatial uniformity of the electrodeposited copper layers. In general, thicker deposits are not necessarily associated with improved microstructural quality, as evidenced by the pronounced dendritic growth observed for some systems. A key observation is the clear discrepancy between edge dendrite height and central dendrite height for deposits obtained from electrolytes containing nicotinic acid, benzotriazole and thiourea. In these cases, dendritic growth is significantly more developed at the sample edges than in the central region, indicating non-uniform current distribution and the presence of edge effects that promote localized growth instabilities. This effect is particularly pronounced for thiourea, which leads to highly unstable deposition characterized by intensive dendritic formation, while benzotriazole and nicotinic acid exhibit intermediate behavior.

In contrast, the electrolyte containing urea provides markedly different results. The dendrite heights measured at the edge and in the central part of the sample are nearly identical, indicating highly uniform growth across the entire surface. The nearly identical edge and central dendrite heights observed for the urea-based electrolyte suggest a significant suppression of edge-related growth instabilities. While urea is not typically classified as a strong leveling agent, its adsorption and complexation effects, combined with flow-assisted mass transport, likely contribute to a more uniform current distribution across the electrode surface.

This observation is fully consistent with the visual inspection of the samples, where no increased dendrite density at the edges is observed for urea-based deposits, unlike in the other cases (Supplementary Materials, Section S2, Figures S1–S4). Additionally, it is worth emphasizing that the trends observed in the micrometric measurements of layer thickness and dendrite height remain consistent with those derived from SEM cross-sectional analysis. This agreement confirms that the applied measurement methodology provides a reliable representation of the macroscopic morphology and supports the interpretation of deposition stability based on dendrite development.

### 3.4. EDS Analysis

**Figure 11:**
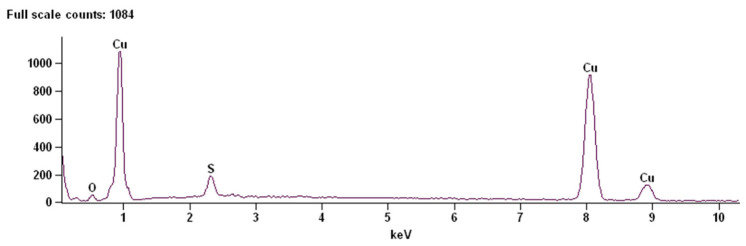
EDS spectrum of the sample deposited from the bath containing nicotinic acid brightener.

Elemental composition of the electrodeposited copper layers obtained from sulfate electroplating baths with different organic brighteners was analyzed using EDS spectroscopy. Figure 11 shows a representative EDS spectrum of the copper layer deposited from the plating bath containing nicotinic acid. EDS spectra of the remaining samples are presented in the Supplementary Materials (Section S3).

The elemental composition (mass fractions, wt.%) obtained from SEM/EDS analyses for each electrodeposited copper surface is presented in Table 3.

In all investigated samples, copper was the dominant element, with mass fractions exceeding 88 wt.%, confirming effective metal deposition under all tested conditions. However, noticeable differences in impurity levels (O and S) were observed depending on the type of brightener, indicating its significant influence on the incorporation of non-metallic species into the deposit.

The sample obtained from the bath containing thiourea exhibited the lowest copper content (88.9 wt.%) and the highest oxygen (6.37 wt.%) and sulfur (4.73 wt.%) contents. This indicates a substantial incorporation of sulfur-containing species and possible formation of copper sulfide or oxide phases. Such behavior is consistent with the strong adsorption and decomposition of thiourea on the cathode surface, leading to the inclusion of reaction by-products within the growing layer. This result correlates well with SEM observations, where thiourea promoted highly irregular, agglomerated and dendritic morphologies, suggesting unstable growth conditions and significant side reactions.

In contrast, deposits obtained with benzotriazole and nicotinic acid showed significantly lower impurity levels. For benzotriazole, the Cu content reached 92.56 wt.% with moderate sulfur incorporation (3.99 wt.%), while nicotinic acid resulted in 92.98 wt.% Cu with comparable oxygen and sulfur contents (~3.5 wt.% each). These results suggest that both additives effectively control surface reactions, limiting excessive incorporation of electrolyte-derived species. Benzotriazole, known for its strong adsorption and corrosion-inhibiting properties, likely forms a protective adsorbed layer that stabilizes deposition and reduces uncontrolled growth. Nicotinic acid, on the other hand, appears to provide a balance between deposition rate and surface stabilization, which is consistent with previously observed relatively smooth and homogeneous morphologies.

The urea-based electrolyte produced the highest copper purity (93.05 wt.%) and the lowest sulfur content (2.61 wt.%). This suggests that urea does not significantly contribute to sulfur incorporation and may act as a mild complexing or leveling agent, promoting relatively clean deposition. However, the slightly elevated oxygen content (4.34 wt.%) may indicate the presence of surface oxides or trapped electrolyte residues. These findings align with morphological observations showing relatively uniform but not perfectly defect-free layers.

Overall, the EDS results clearly demonstrate that the choice of brightener strongly influences not only the morphology but also the chemical purity of electrodeposited copper layers. Sulfur-containing additives such as thiourea lead to significant contamination and unstable growth, while nitrogen-based additives like benzotriazole, nicotinic acid and urea provide improved control over the deposition process. Among them, urea and nicotinic acid appear particularly promising for achieving a favorable balance between high copper content and limited impurity incorporation.

These compositional trends are consistent with the previously discussed SEM observations and confirm that the incorporation of non-metallic elements can serve as an important indicator of deposition stability and layer quality. In particular, the increased oxygen and sulfur contents correlate with dendritic growth and structural defects, supporting the use of elemental analysis as a complementary tool for process optimization in electrodeposition-based 3D printing systems.

## 4. Conclusions

A comparative evaluation of the investigated additives reveals distinct differences in their mechanisms of action and suitability for electrochemical additive manufacturing of copper. Nicotinic acid, benzotriazole, thiourea and urea exhibit progressively different levels of surface activity, ranging from weak interaction with the electrode interface to strong adsorption and complex surface reactions. Overall, the additives can be ranked in terms of their functional role in electrochemical 3D printing as follows: nicotinic acid as an optimal process-modifying additive (optimal working concentration range: 0.1–0.6 mol/L), benzotriazole as a strong inhibitor requiring careful optimization (optimal working concentration range: 0.001–0.01 mol/L), thiourea as a potentially destabilizing component (not recommended for use in electrochemical 3D printing) and urea as a stabilizing or supporting additive (broad working concentration range: 0.05–1.00 mol/L). This classification provides a useful framework for designing multi-component electroplating baths tailored for controlled, layer-by-layer copper deposition.

The SEM and EDS analyses demonstrated that the type of organic additive strongly influences the morphology, stability, uniformity and chemical purity of copper electrodeposition from sulfate baths. Thiourea caused highly unstable growth with excessive dendritic formation and significant contamination of the deposits, whereas benzotriazole promoted grain refinement but did not fully suppress vertical growth instabilities. Nicotinic acid enabled relatively thick deposits with moderate dendritic contribution. In contrast, urea provided the most stable deposition conditions, yielding compact, uniform copper layers with minimal dendritic growth, effective suppression of edge effects and the highest copper purity. The dendrite height-to-layer thickness ratio proved to be a useful quantitative parameter for evaluating deposition stability. Overall, the results confirm that appropriate additive selection plays a critical role in optimizing copper electrodeposition processes, particularly for flow-assisted and electrochemical additive manufacturing applications.

## Figures and Tables

**Table 1:** Compositions of analyzed electroplating baths and electrodeposition potentials used.

No.	CuSO_4_ Concentration	H_2_SO_4_ Concentration [mol/dm^3^]	Brightening Agent		Potential (vs. Ag/AgCl) [V]
			Name	C [mol/dm^3^]	
1	saturated	1	Nicotinic acid	0.200	−0.45
2	saturated	1	Benzotriazole	0.005	−0.55
3	saturated	1	Thiourea	0.020	−0.40
4	saturated	1	Urea	0.040	−0.25

**Table 2:** Comparison of layer thickness and dendrite height in cross-sections of copper samples electrodeposited from electroplating baths with different brighteners, where t_Cu_—cross-sectional thickness of the electrodeposited copper layers [mm] and h_d_—dendrite heights [mm].

Brightener	t_Cu_ [mm]	h_d_ [mm]	h_d_/t_Cu_ [-]
Nicotinic acid	2.37 ± 0.04	0.84 ± 0.16	0.35 ± 0.07
Benzotriazole	1.78 ± 0.02	1.15 ± 0.14	0.65 ± 0.08
Thiourea	1.34 ± 0.11	1.78 ± 0.26	1.33 ± 0.29
Urea	1.79 ± 0.08	0.21 ± 0.03	0.12 ± 0.02

**Table 3:** Elemental composition of the analyzed electrodeposited copper layers.

Brightener	Elemental Mass Fraction [wt.%]		
	Cu	O	S
Nicotinic acid	92.98	3.48	3.54
Benzotriazole	92.56	3.45	3.99
Thiourea	88.9	6.37	4.73
Urea	93.05	4.34	2.61

## Data Availability

The original contributions presented in this study are included in the article/Supplementary Material. Further inquiries can be directed to the corresponding author.

## Supplementary Materials

The following supporting information can be downloaded at: https://www.mdpi.com/article/10.3390/molecules31101635/s1, Table S1. Compositions of electroplating baths containing nicotinic acid, used in linear voltammetry tests. Table S2. Compositions of electroplating baths containing **benzotriazole**, used in linear voltammetry tests. Table S3. Compositions of electroplating baths containing **thiourea**, used in linear voltammetry tests. Table S4. Compositions of electroplating baths containing **urea**, used in linear voltammetry tests. Figure S1. Images of the surfaces of electrodeposited copper layers obtained using electroplating baths with **nicotinic acid** as a brightening agent. Figure S2. Images of the surfaces of electrodeposited copper layers obtained using electroplating baths with **benzotriazole** as a brightening agent. Figure S3. Images of the surfaces of electrodeposited copper layers obtained using electroplating baths with **thiourea** as a brightening agent. Figure S4. Images of the surfaces of electrodeposited copper layers obtained using electroplating baths with **urea** as a brightening agent. Figure S5. EDS spectrum of the sample electrodeposited from the bath containing benzotriazole acid as a brightener additive. Figure S6. EDS spectrum of the sample electrodeposited from the bath containing thiourea acid as a brightener additive. Figure S7. EDS spectrum of the sample electrodeposited from the bath containing urea acid as a brightener additive. Figure S8. Experimental setup for copper electrodeposition in flow mode simulating the conditions of copper 3D printing by electrodeposition (A), the working area (B) and the electrode arrangement (C) in the electroplating bath (1—programmable DC power supply with potentiostat, 2—peristaltic pump, 3—electroplating bath container, 4—inlet hose, 5—outlet hose, 6—cathode mounting head, 7—ball joint, 8—Ag/AgCl reference electrode, 9—Pt anode, 10—Cu cathode, 11—electrodeposited copper layer). Section S1. Composition of samples used for linear sweep voltammetry measurements. Section S2. Optical microscopy. Section S3. EDS spectra. Section S4. Electrodeposition experimental setup. Section S5. Selection of brightener concentrations and electrodeposition potentials. References [42,43,44,45,46] are cited in the Supplementary Materials.
