# Supplementary material for: Role of Selected Organic Additives in Sulfate-Based Electroplating Baths for Copper Electrodeposition Toward Additive Manufacturing
Source: Molecules. 2026 May 13;31(10):1635. doi: 10.3390/molecules31101635 (PMC13209522; doi:10.3390/molecules31101635)
Supplement: Supplementary file 1 [file molecules-31-01635-s001.zip › molecules-4304001-supplementary.pdf]

Supplementary Materials

Role of Selected Organic Additives in Sulfate-Based Electroplating Baths for Copper Electrodeposition Toward Additive Manufacturing

Dawid Kiesiewicz [1], Karolina Syrek [2], Paweł Niezgoda [3] and Maciej Pilch [1,*]

1 Faculty of Civil Engineering, Cracow University of Technology, Warszawska 24, 31-155 Cracow, Poland
2 Faculty of Chemistry, Jagiellonian University, Gronostajowa 2, 30-387 Cracow, Poland; syrek@chemia.uj.edu.pl
3 Faculty of Chemical Engineering and Technology, Cracow University of Technology, Warszawska 24, 31-155 Cracow, Poland
* Correspondence: maciej.pilch@pk.edu.pl; Tel.: +48-12-628-35-70

1) Section S1. Composition of samples used for linear sweep voltammetry measurements.

The compositions of the investigated electroplating baths are summarized in Tables S1-S4.

Table S1. Compositions of electroplating baths containing **nicotinic acid**, used in linear voltammetry tests.

**Sample no.**	**$CuSO_4$**	**H_2SO_4 [mol/L]**	**Brightener concentration [mol/L]**
1	saturated	1	0.00
2			0.01
3			0.02
4			0.03
5			0.04
6			0.05
7			0.06
8			0.07
9			0.08
10			0.09
11			0.20
12			0.30
13			0.40
14			0.50
15			0.60
16			0.70
17			0.80
18			0.90
19			1.00

Table S2. Compositions of electroplating baths containing **benzotriazole**, used in linear voltammetry tests.

**Sample no.**	**CuSO4**	**H_2SO_4 [mol/L]**	**Brightener concentration [mol/L]**
1	saturated	1	0.000
2			0.001
3			0.002
4			0.003
5			0.004
6			0.005
7			0.006
8			0.007
9			0.008
10			0.009
11			0.015
12			0.020
13			0.030
14			0.040
15			0.050
16			0.060
17			0.070
18			0.080
19			0.090
20			0.100

Table S3. Compositions of electroplating baths containing **thiourea**, used in linear voltammetry tests.

**Sample no.**	**$CuSO_4$**	**H_2SO_4 [mol/L]**	**Brightener concentration [mol/L]**
1	saturated	1	0.000
2			0.001
3			0.002
4			0.003
5			0.004
6			0.005
7			0.006
8			0.007
9			0.008
10			0.009
11			0.020
12			0.030
13			0.040
14			0.050
15			0.060
16			0.070
17			0.080
18			0.090
19			0.100

Table S4. Compositions of electroplating baths containing **urea**, used in linear voltammetry tests.

Sample no.	$CuSO_4$	H_2SO_4 [mol/L]	Brightener concentration [mol/L]
1	saturated	1	0.00
2			0.01
3			0.02
4			0.03
5			0.04
6			0.05
7			0.06
8			0.07
9			0.08
10			0.09
11			0.20
12			0.30
13			0.40
14			0.50
15			0.60
16			0.70
17			0.80
18			0.90
19			1.00

2) Section S2. Optical microscopy

Photographs of copper layers electrodeposited from electroplating baths containing different brighteners are presented in Figures S1–S4.

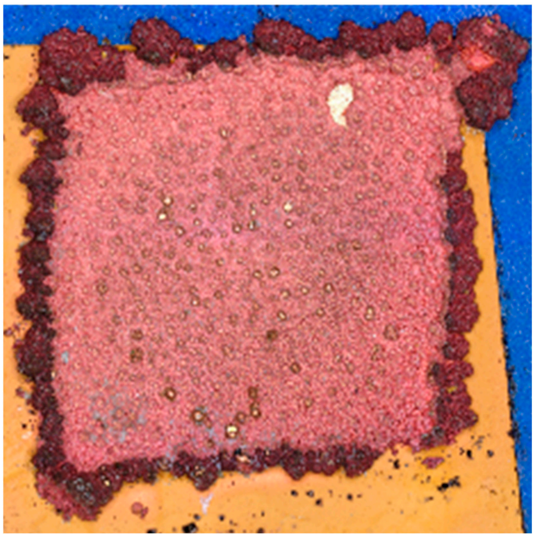

Figure S1. Images of the surfaces of electrodeposited copper layers obtained using electroplating baths with **nicotinic acid** as a brightening agent.

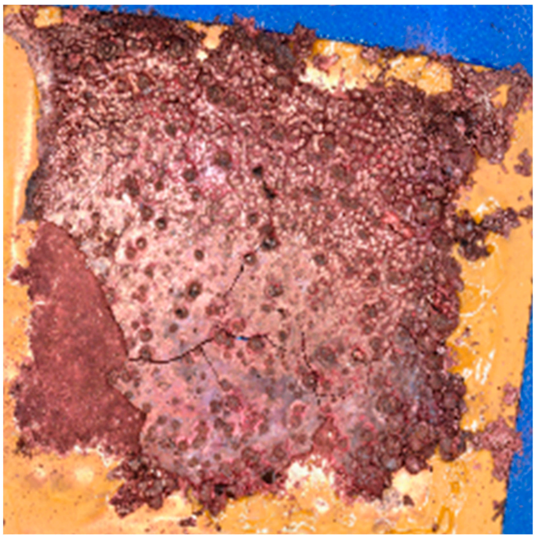

Figure S2. Images of the surfaces of electrodeposited copper layers obtained using electroplating baths with **benzotriazole** as a brightening agent.

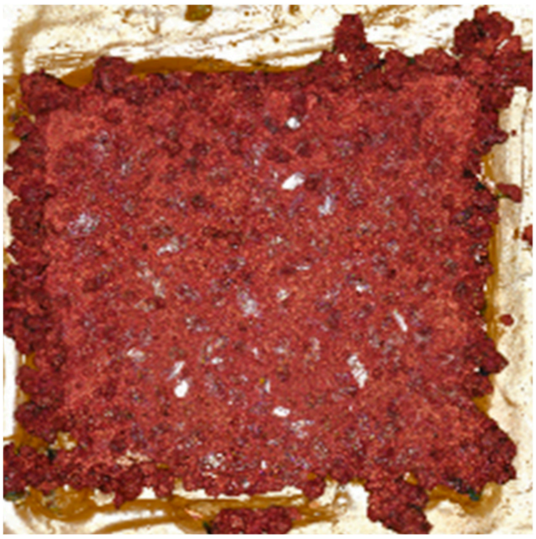

Figure S3. Images of the surfaces of electrodeposited copper layers obtained using electroplating baths with **thiourea** as a brightening agent.

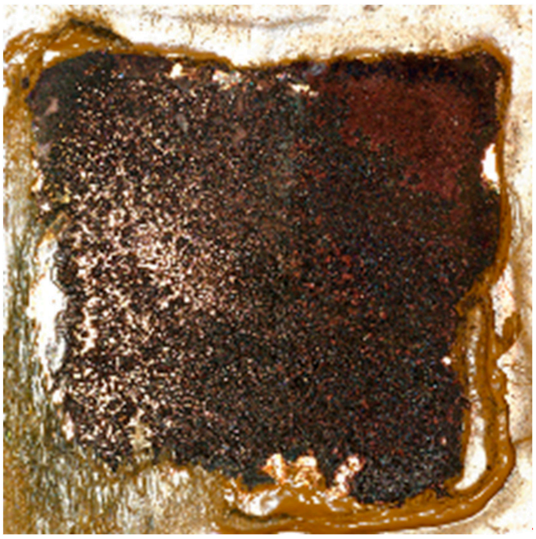

Figure S4. Images of the surfaces of electrodeposited copper layers obtained using electroplating baths with **urea** as a brightening agent.

3) **Section S3. EDS spectra**

EDS spectra of the surfaces of copper layers electrodeposited from electroplating baths containing different brighteners are presented in Figures S5–S7.

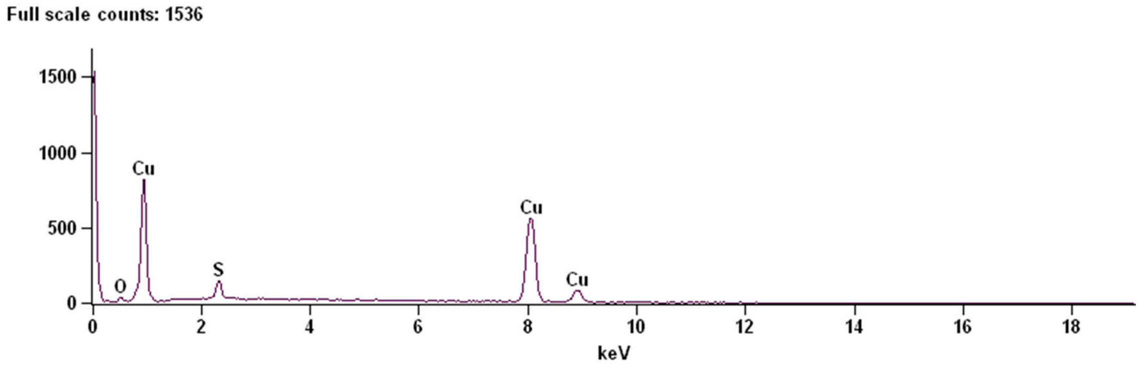

Figure S5. EDS spectrum of the sample electrodeposited from the bath containing benzotriazole acid as a brightener additive.

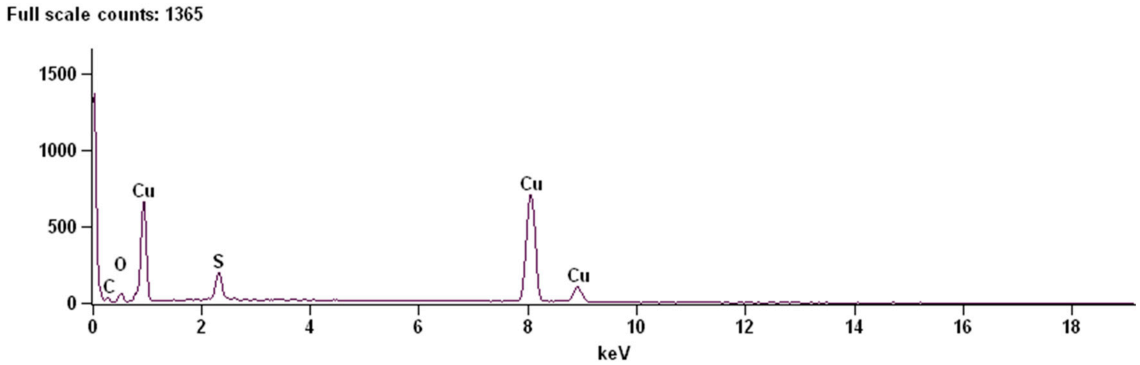

Figure S6. EDS spectrum of the sample electrodeposited from the bath containing thiourea acid as a brightener additive.

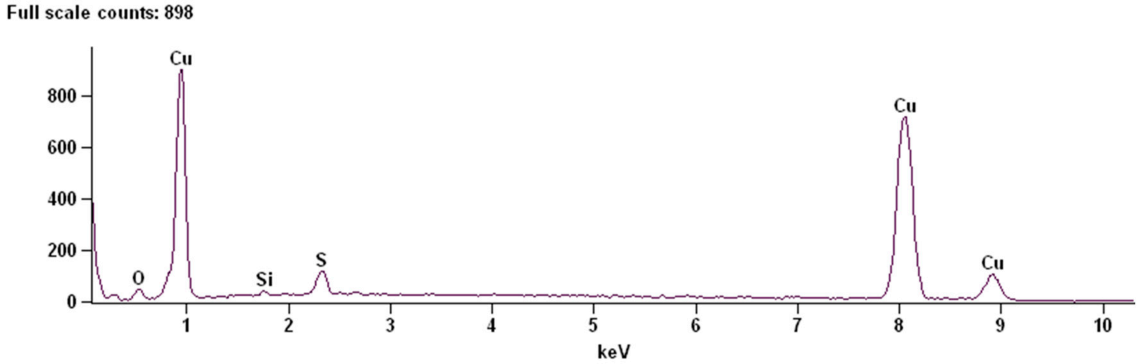

Figure S7. EDS spectrum of the sample electrodeposited from the bath containing urea acid as a brightener additive.

4) Section S4. Electrodeposition experimental setup

Photographs of the experimental setup for copper electrodeposition in flow mode, simulating the conditions of copper 3D printing by electrodeposition, are presented in Figure S8.

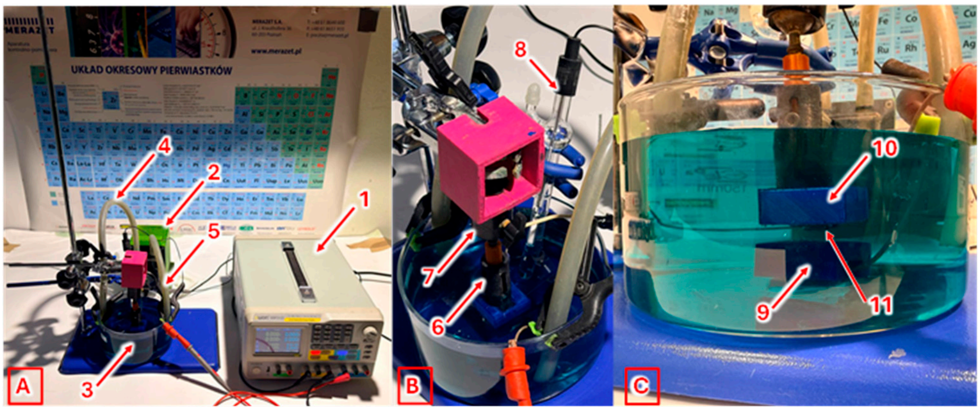

Figure S8. Experimental setup for copper electrodeposition in flow mode simulating the conditions of copper 3D printing by electrodeposition (A), the working area (B) and the electrode arrangement (C) in the electroplating bath (1 – programmable DC power supply with potentiostat, 2 – peristaltic pump, 3 – electroplating bath container, 4 – inlet hose, 5 – outlet hose, 6 – cathode mounting head, 7 – ball joint, 8 – Ag/AgCl reference electrode, 9 – Pt anode, 10 – Cu cathode, 11 - electrodeposited copper layer) [1].

5) Section S5. Selection of brightener concentrations and electrodeposition potentials

Brightener concentrations and electrodeposition potentials were selected individually for each type of electroplating bath analyzed.

For the electrodeposition of test copper layers from different electroplating baths, the bath compositions were selected, based on the voltammetric analysis (Section 3.3 of the main manuscript), at brightener concentrations near the midpoint of the ranges in which the cyclic voltammograms remain approximately unchanged with small variations in brightener concentration. A weak dependence of the voltammetric response on the brightener concentration indicates that the electrodeposition process is not strongly controlled by additive adsorption, which is beneficial from the perspective of process stability. Under such conditions, local fluctuations in additive concentration during electrodeposition are less likely to induce significant variations in current density, thereby promoting more uniform layer growth. This interpretation is valid for electroplating baths containing nicotinic acid, urea and thiourea additives. Specifically, as shown in Figures 2, 6 and 8 (Section 3.1 of the main manuscript), for electroplating baths containing nicotinic acid, urea and thiourea, concentration ranges can be identified in which a small change in additive concentration results in both the cathodic signal potential and the cathodic signal current remaining approximately constant or changing significantly less than for the same concentration variation outside these concentration ranges. For this reason, these concentration ranges were selected as the recommended ranges for the three aforementioned additives.

The selection of the benzotriazole concentration range (below 0.01 M) for the electrodeposition process was based directly on the voltammetric results presented in Figure 3 (Section 3.1 of the main manuscript). As can be observed from the voltammograms, although even at low BTA concentrations (below 0.009 M) an increase in BTA concentration causes a decrease in the cathodic signal potential, increasing the BTA concentration above 0.009 M leads to a clear shift of the cathodic

response toward more negative potentials, into the potential range below approximately −0.6 V vs. Ag/AgCl, accompanied by a noticeable decrease in cathodic current. For BTA concentrations higher than 0.009 M, a significant increase in cathodic current is observed only at potentials more negative than approximately −0.6 V vs. Ag/AgCl. Additionally, for BTA concentrations higher than 0.02 M, the copper reduction signal is strongly suppressed within the investigated potential range. In this case, no well-defined cathodic extremum characteristic of copper reduction is observed in the voltammograms. In contrast, for BTA concentrations significantly lower than 0.009 M, the cathodic current increases (the signal corresponding to copper ion reduction appears) at less negative potentials and reaches higher values within the same potential range. Based on these observations and maintaining a safety margin, the BTA concentration range below 0.01 M was selected as the recommended range for the electrodeposition process, since concentrations above 0.01 M shift the copper reduction process toward more negative potentials below approximately −0.6 V vs. Ag/AgCl, reduce the current values within the investigated range and lead to the disappearance of the well-defined cathodic extremum characteristic of copper reduction. The selected concentration range (<0.01 M) ensures that the cathodic process occurs within a less negative potential range and with a higher current response under comparable conditions.

Similarly, the electrodeposition potentials were chosen, based on the voltammetric analysis (Section 3.3 of the main manuscript), on the positive side of the extremum corresponding to copper reduction in the voltammogram for a given bath with the previously selected brightener concentration. The optimal deposition potential should be selected not at the current minimum nor in the region of more negative potentials beyond it, but rather in the vicinity of the onset of intensive copper reduction or slightly on the positive side of the extremum. Although more negative potentials yield higher current densities, they increasingly promote mass transport limitations, local depletion of Cu^{2+} ions, edge effects and dendritic growth, whereas slightly less negative potentials provide a better balance between deposition rate and surface uniformity. The same criterion remains valid under flow conditions, although the enhanced mass transport allows operation at more negative potentials without immediate onset of diffusion limitations. Nevertheless, at sufficiently high overpotentials, local Cu^{2+} depletion, current density nonuniformities and edge effects still promote morphological instabilities, making slightly less negative potentials preferable for achieving a balance between deposition rate and surface uniformity. Additionally, it should be noted that the Cu^{2+} reduction signal in the voltammogram is shifted toward more negative potentials for a carbon electrode compared to a copper electrode. This is due to the need to initiate copper nucleation on a foreign substrate, which involves a higher energy barrier and greater overpotential. In contrast, on a copper electrode, the process proceeds as growth on the same metal and therefore occurs at less negative potentials. For this reason, the electrodeposition potentials were selected with a safety margin. This ensures that, under real conditions, the process is not carried out in the region of very high cathodic currents, but rather near the potential corresponding to the voltammetric reduction peak of copper under these conditions [2-5].

References:

1. Kiesiewicz, D.; Syrek, K.; Niezgoda, P.; Żydowski, S.; Łagan, S.; Pilch, M. Initial Study of Feedstock Material Compositions for 3D Printing of Hybrid Metal–Polymer Components via Electrodeposition and Photopolymerization in an Electroplating Bath Environment. Molecules 2026, 31, 1316. DOI: 10.3390/molecules31081316.

2. Gunawardena, G.; Hills, G.; Montenegro, M.I.; Scharifker, B.R. Electrochemical nucleation: Part IV. Electrodeposition of copper onto vitreous carbon. J. Electroanal. Chem. Interfacial Electrochem. 1985, 184, 357–367. DOI: 10.1016/0368-1874(85)85539-8.
3. Jaya, S.; Prasada Rao, T.; Prabhakara Rao, G. Electrochemical phase formation—I. The electrodeposition of copper on glassy carbon. Electrochim. Acta 1986, 31, 343–348. DOI: 10.1016/0013-4686(86)80088-3.
4. Grujicic, D.; Pesic, B. Electrodeposition of copper: The nucleation mechanisms. Electrochim. Acta 2002, 47, 2901–2912. DOI: 10.1016/S0013-4686(02)00161-5.
5. Danilov, A.I.; Molodkina, E.B.; Polukarov, Yu.M. Initial stages of copper electrocrystallization on a glassy-carbon ring–disk electrode from sulfate electrolytes of various acidity: A cyclic voltammetry study. Russ. J. Electrochem. 2002, 38, 732–742. DOI: 10.1023/A:1016392515937.
